# Predicting operative mortality in octogenarians for isolated coronary artery bypass grafting surgery: a retrospective study

**DOI:** 10.1186/s12872-017-0706-z

**Published:** 2017-11-02

**Authors:** Jessica G. Y. Luc, Michelle M. Graham, Colleen M. Norris, Sadek Al Shouli, Yugmel S. Nijjar, Steven R. Meyer

**Affiliations:** 1 Division of Cardiac Surgery, Department of Surgery, Faculty of Medicine and Dentistry, University of Alberta, Edmonton, Canada; 2 Mazankowski Alberta Heart Institute, Edmonton, Canada; 3 Division of Cardiology, Department of Medicine, Faculty of Medicine and Dentistry, University of Alberta, Edmonton, Canada

**Keywords:** Cardiovascular research, Coronary artery disease, Risk prediction, Octogenarians, Cardiovascular Surgery, Coronary Artery Disease, Risk Stratification

## Abstract

**Background:**

Available cardiac surgery risk scores have not been validated in octogenarians. Our objective was to compare the predictive ability of the Society of Thoracic Surgeons (STS) score, EuroSCORE I, and EuroSCORE II in elderly patients undergoing isolated coronary artery bypass grafting surgery (CABG).

**Methods:**

All patients who underwent isolated CABG (2002 – 2008) were identified from the Alberta Provincial Project for Outcomes Assessment in Coronary Heart Disease (APPROACH) registry. All patients aged 80 and older (*n* = 304) were then matched 1:2 with a randomly selected control group of patients under age 80 (*n* = 608 of 4732). Risk scores were calculated. Discriminatory accuracy of the risk models was assessed by plotting the areas under the receiver operator characteristic (AUC) and comparing the observed to predicted operative mortality.

**Results:**

Octogenarians had a significantly higher predicted mortality by STS Score (3 ± 2% vs. 1 ± 1%; *p* < 0.001), additive EuroSCORE (8 ± 3% vs. 4 ± 3%; p < 0.001), logistic EuroSCORE (15 ± 14% vs. 5 ± 6%; p < 0.001), and EuroSCORE II (4 ± 3% vs. 2 ± 2%; p < 0.001) compared to patients under age 80 years. Observed mortality was 2% and 1% for patients age 80 and older and under age 80, respectively (*p* = 0.323). AUC revealed areas for STS, additive and logistic EuroSCORE I and EuroSCORE II, respectively, for patients age 80 and older (0.671, 0.709, 0.694, 0.794) and under age 80 (0.829, 0.750, 0.785, 0.845).

**Conclusion:**

All risk prediction models assessed overestimated surgical risk, particularly in octogenarians. EuroSCORE II demonstrated better discriminatory accuracy in this population. Inclusion of new variables into these risk models, such as frailty, may allow for more accurate prediction of true operative risk.

## Background

Coronary artery disease is a leading cause of morbidity and mortality in octogenarian patients [1, 2]. Octogenarians represent the fastest growing segment of our population; over 40% of the very elderly manifest cardiovascular disease [3]. The indications for coronary artery bypass grafting (CABG) surgery are well defined and the number of octogenarians being referred for surgical coronary revascularization is increasing [4]. Accurate risk stratification and the prediction of operative mortality is essential as this aids the clinical decision-making process, helps estimate the need for resources, facilitates proper patient counseling, informed consent, and allows for monitoring of surgeon and institution performance through risk-adjusted outcomes.

Risk-scoring algorithms based on both patient history and functional status have been established. The most widely used are the Society of Thoracic Surgeons Predicted Risk of Mortality (STS) score and the European System for Cardiac Operative Risk Evaluation (EuroSCORE) [5, 6]. Despite the acceptance of these risk scores in the context of CABG, there is little data on the performance of the revised EuroSCORE II in comparison to the established STS, additive EuroSCORE and the logistic Euroscore. Furthermore, these scores have not been validated to specifically predict operative mortality in octogenarians.

The aim of the current study was to evaluate and compare the performance of the recently introduced EuroSCORE II with its previous version EuroSCORE (additive and logistic) and the STS score in predicting perioperative mortality in patients age 80 and older undergoing isolated CABG at our institution.

## Methods

### Patients and procedures

We retrospectively reviewed 304 consecutive patients age 80 and older and a randomly selected cohort of adult patients under age 80 (*n* = 608 of 4732) who underwent isolated CABG between January 2002 and December 2008 at the University of Alberta. The older group was compared in a 1:2 ratio to the younger group. Patients undergoing concomitant surgery (e.g., valve, vascular, or congenital) were excluded. All non-emergent cardiac surgery cases undergo multidisciplinary review at our institution. These multidisciplinary rounds include cardiac surgeons, interventional cardiologists and cardiologists. A comparison ratio of 1:2 was selected instead of 1:1 as our population of elderly patients is modest; a larger control group of younger patients generates a more normally distributed dataset for analysis.

The Alberta Provincial Project for Outcome Assessment in Coronary Heart Disease (APPROACH) database has been previously described [7]. In brief, APPROACH is a prospective clinical data collection initiative capturing all patients undergoing cardiac catheterization or cardiac surgery in the province of Alberta, Canada since 1995. The registry includes detailed individual patient demographic, medical, angiographic, surgical and postoperative information. Preoperative patient demographic and medical variables including risk factors, comorbidities, cardiac diagnostic and surgical procedural information are entered into the dataset. Patients captured by the registry are longitudinally followed for all cardiac-specific investigations, interventions and outcomes. Mortality is tracked through an Alberta Bureau of Vital Statistics data linkage [8]. The University of Alberta Health Research Ethics Board has approved the waiver of patient consent for this data registry (Pro00042669).

The APPROACH database was queried for pre, intra and postoperative data of the patients in this study. Risk scores were then calculated online using the official websites and calculators (STS score: http://riskcalc.sts.org/stswebriskcalc/#/calculate; additive and logistic EuroSCORE: http://euroscore.org/calcold.html; EuroSCORE II: http://euroscore.org/calc.html). The simple additive EuroSCORE and the full logistic EuroSCORE, were included because both have been adopted in clinical practice for reasons of simplicity for the additive model and accuracy for the logistic model [9].

### Outcomes of interest

The primary outcome of interest was perioperative mortality, defined as any death that occurred during the initial hospitalization or within 30 days of isolated CABG. The expected 30-day mortality based on the calculated STS score, EuroSCORE (additive and logistic), and EuroSCORE II were compared with the observed 30-day mortality.

### Statistical analysis

Continuous variables were described as mean and standard deviation and categorical variables as percentages. Comparisons between continuous data were made with student t-test or Mann-Whitney U test and comparisons between categorical data were made with Chi-square or Fisher’s exact test where appropriate. Sensitivity and specificity of expected versus observed mortality were summarized by receiver operator curves and the area under the receiver operator characteristic (AUC). Net reclassification improvement (NRI) analysis was also performed to formally assess the various scores accuracy in predicting risk in both age groups. A *p*-value of less than 0.05 was considered significant. All statistical analyses were performed using Statistical Package for Social Sciences (SPSS Statistics, version 21, Chicago, Illinois).

## Results

From January 1, 2002 to December 31, 2008, a total of 4732 patients underwent isolated CABG at the University of Alberta. Of these, 304 patients were aged 80 years or older. Baseline characteristics are outlined in Table 1. In general, elderly patients had more comorbidities, cardiac risk factors and were more likely to undergo emergency surgery. Use of multiple arterial conduits is rare in Alberta. No patients in this octogenarian cohort received a third arterial graft.

**Table 1:** Baseline Demographics

Variable	Under Age 80 (*n* = 608)	Age 80 and Older(*n* = 304)	*P Value*
Age	63.8	82.1	<0.001^¶^
Gender (Female)	92 (15.1)	78 (25.7)	0.001^¶^
Body Surface Area	1.94 ± 0.17	1.86 ± 0.18	<0.001^¶^
Weight (kg)	80.7 ± 12.5	74.8 ± 11.9	<0.001^¶^
Renal Impairment	116 (19.0)	55 (18.0)	0.044^¶^
Hypertension	452 (74.3)	243 (79.9)	0.062
Hypercholesterolemia	519 (85.4)	211 (69.4)	0.839
Diabetes	204 (33.6)	76 (25.0)	0.008^¶^
Extracardiac Arteriopathy	48 (7.9)	31 (10.2)	0.244
Reduced Left Ventricular Ejection Fraction	340 (55.9)	137 (45.1)	0.001^¶^
Cerebrovascular Disease	44 (7.2)	40 (13.2)	<0.001^¶^
Chronic Obstructive Pulmonary Disease	65 (10.7)	41 (13.5)	<0.001^¶^
Emergency Surgery	16 (2.6)	12 (3.9)	0.001^¶^

Continuous data are reported as mean ± standard deviation; categorical data are (%) are presented by frequency. ^¶^ Indicates significance *p* <V 0.05

**Fig. 1:**
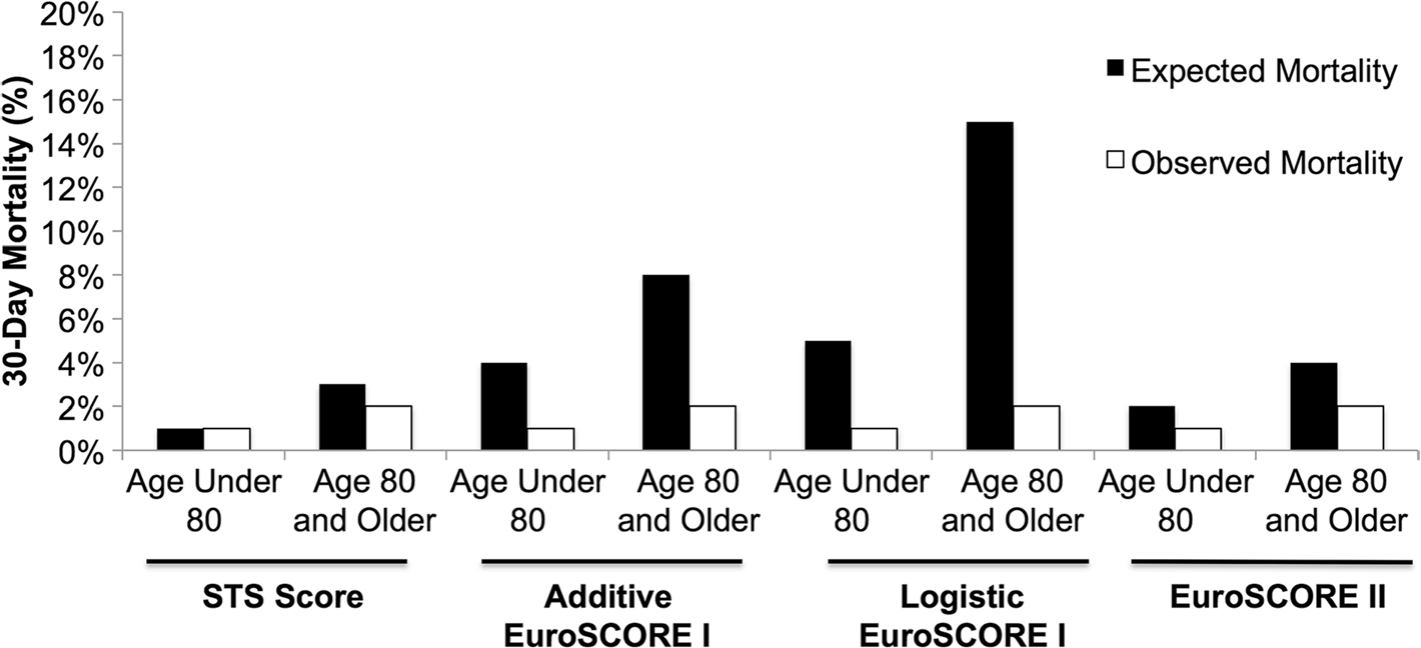
STS risk score, additive EuroSCORE, logistic EuroSCORE, and EuroSCORE II overestimates operative mortality in patients age 80 and older. Expected 30-day mortality was compared to observed 30-day mortality in patients under the age of 80 and patients age 80 and older who underwent isolated CABG

Relative to younger patients, those aged 80 and older had a significantly higher mean predicted mortality by the STS Score (3 ± 2% vs. 1 ± 1%; *p* < 0.001), additive EuroSCORE (8 ± 3% vs. 4 ± 3%; p < 0.001), logistic EuroSCORE (15 ± 14% vs. 5 ± 6%; p < 0.001), and EuroSCORE II (4 ± 3% vs. 2 ± 2%; p < 0.001) (Fig. 1). The total observed perioperative mortality of 2% for patients aged 80 years and older was not significantly different from the observed perioperative mortality of 1% for patients under age 80 (*p* = 0.323).

**Fig. 2:**
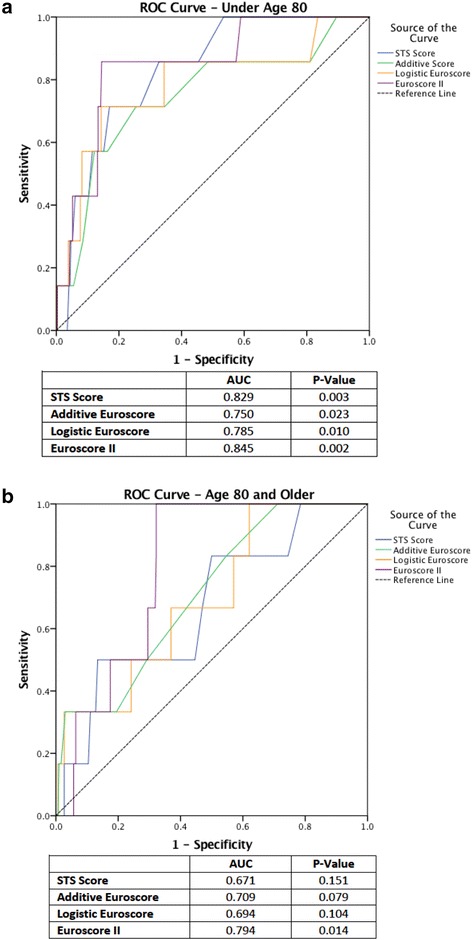
EuroSCORE II was superior to STS and EuroSCORE I in predicting operative mortality for both patients under and over the age of 80. Receiver operator characteristic curve for STS, additive EuroSCORE, logistic EuroSCORE and EuroSCORE II predicted risk of operative mortality in patients (**a**) under age 80 and (**b**) age 80 and older

The receiver operator characteristic (ROC) for both patient groups are shown in Fig. 2a and b respectively. Patient’s age 80 and older had an AUC for the STS score of (0.672; *p* = 0.151, 95% confidence interval [CI] 0.459 to 0.883), additive EuroSCORE (0.709; *p* = 0.079, 95% CI 0.523 to 0.896), logistic EuroSCORE (0.694; *p* = 0.104, 95% CI 0.499 to 0.889) and EuroSCORE II (0.794; *p* = 0.014, 95% CI 0.696 to 0.893). Patients under age 80 had an AUC for the STS score of (0.829; *p* = 0.003, 95% CI 0.710 to 0.947), additive EuroSCORE (0.750; *p* = 0.023, 95% CI 0.544 to 0.955), logistic EuroSCORE (0.785; *p* = 0.010, 95% CI 0.585 to 0.985) and EuroSCORE II (0.845; *p* = 0.002, 95% CI 0.709 to 0.980). The *p*-value under the AUC represents the ability and accuracy of the score in predicting perioperative mortality in patients who undergo CABG surgery. Overall, all scores assessed underperformed patients age 80 and older compared, with EuroSCORE II demonstrating the best predictive ability in this group.

The net reclassification improvement index (NRI) of STS compared to EuroSCORE II was calculated. Area under the curve analysis demonstrates that the areas of the three curves are similar for patients under age 80, but different for patients age 80 and over where EuroSCORE II had the largest area. The Delong test indicates that the difference of the AUC between the three curves is not significant for both groups. However, NRI analysis reveals that EuroSCORE adds 16% reclassification improvement compared to STS for age under 80, and 67% improvement for in those age 80 and older. The 67% improvement results from ability of EuroSCORE II to discriminate all 6 deaths, while STS is able to discriminate only half (3 out of 6) of deaths.

## Discussion

We demonstrate in a large series of consecutive octogenarians undergoing isolated CABG that EuroSCORE II had the best performance for risk assessment among the tools available. However, the expected mortality calculated by all four risk scores overestimated perioperative risk for patients of all ages, most particularly in octogenarians.

Our study is unique in that it includes all four risk scores with a focused evaluation of their performance in isolated CABG surgery. This is particularly relevant given that octogenarians represent a growing proportion of patients being referred for CABG surgery [10]. Early studies report cardiac surgery operative mortality rates of up to 11% [11, 12]. Refinements in surgical technique and perioperative management has resulted in significantly improved outcomes [13], as well, differences in observed operative mortality in other reports may stem from inherent country or population specific outcomes [14, 15]. We report a low observed perioperative mortality of 2% in patients age 80 and older which is not different from existing contemporary reports [4, 16, 17]. Although elderly patients have worse early outcomes compared to younger patients, long-term outcomes for octogenarians at our institution after CABG are similar to, if not better than, the age-adjusted Canadian population. These are demonstrated by previously published reports on our cohort examining intermediate and long-term survival post-operatively for octogenarians compared to younger subjects [4, 18, 19].

Accurate risk stratification facilitates improved patient selection, prevents complications [20], improves patient quality of life and can justify the cost effectiveness of the procedure [21]. However, risk models, although helpful and objective for operative risk assessment and clinical decision-making, are not without their limitations. The original EuroSCORE and EuroSCORE II were developed with a patient population mean age of 62.5 years and 64.9 years, respectively [13, 22]. Our results show that all scores assessed significantly overestimated operative risk in octogenarians, and were well calibrated in patients under age 80. This identified imprecision in operative risk prediction has important implications for clinical decision making in elderly patients.

Frailty has been shown to be an independent predictor for both postoperative complications and in-hospital mortality after adjusting for age [23]. It is estimated that 20% of octogenarians are frail [24]. Although considered difficult to quantify as compared to the comorbidity-based risk assessment models currently in use, several scores have emerged as an attempt to assess frailty including the Edmonton Frailty Scale, Comprehensive Assessment of Frailty test, Fried Frailty Scale and others [24–26] These frailty scores are a multidimensional assessment of health and functional status incorporating sociodemographic, biomedical, cognitive capacity, independence with daily activities, social support and mood [27, 28].

Incorporating a quantitative measure of the degree of frailty can introduce objectivity to the assessment of biological status to complement conventional comorbidity risk assessment and improve the accuracy of operative risk assessment in the elderly. This would allow more accurate stratification of risk than current models and aid in selecting the most appropriate modality of intervention to best optimize outcomes for patients. In an age 70 and older population, Prudon et al. [29] demonstrated that the addition of gait speed to logistic EuroSCORE improved the accuracy of the model. Sundermann et al. [24] used a frailty score in addition to conventional risk scores and found frailty to only moderately correlate with STS and EuroSCORE risk models. Other investigators have demonstrated the utility of a frailty score in the preoperative assessment of their patients [24, 30]. Afilalo et al. demonstrate that both frailty and disability (as defined by a 5-m gait speed ≥ 6 seconds and the presence of 3 or more impairments on the Nagi scale) are complementary and additive to existing risk scores where inclusion of them improved the discrimination of the STS score [30]. Thus, incorporation of various risk factors specific to the elderly population as measures of physiological vulnerability may improve the effectiveness of cardiac surgical risk models [31].

Frailty is an emerging concept; collaborative efforts towards defining the optimal method towards measuring frailty and disability deserve further exploration. Multidisciplinary teams involving cardiac surgery, cardiology, geriatric medicine, physiotherapy and occupational therapy may help address the diverse elements specific to the elderly that contribute to increased risk [32]. A 64-slice-coronary computed tomography shows promise as a non-invasive alternative to the standard coronary angiography to detect CABG graft stenosis [33]. However, in a frail octogenarian population, symptoms and quality of life should be the driver for any further invasive or non-invasive investigations.

### Limitations

There are several limitations to this study. First, this is a single institution study, thus our results may not apply to those from other institutions and countries. Second, the present study only included patients undergoing isolated CABG and the results cannot be extrapolated to patients undergoing other cardiac surgical procedures. Third, EuroSCORE II has introduced two variables, poor mobility and pulmonary artery pressure, which are not consistently recorded in the APPROACH database; therefore all patients were analyzed as not scoring for this risk factor. However, if these risks were present in our entire elderly cohort, the estimated risk of surgery would have further increased. Finally, our intention was neither to develop a new score nor to investigate the impact of individual variables on specific postoperative complications. While the predictive value for mortality differs considerably from that of morbidity for most of the variables included in the risk models [34], our analysis focused on the discriminatory ability of these scores in predicting perioperative mortality – which was what these risk scores were originally designed to achieve.

## Conclusion

Currently, all risk prediction models overestimate surgical risk, particularly in octogenarians. The EuroSCORE II demonstrated better discriminatory accuracy for predicting operative mortality than STS, additive and logistic EuroSCORE in this population. Inclusion of new variables into these risk models, such as frailty, that are of particular interest in the elderly, may allow for more accurate prediction of true operative mortality and thus lead to improved decision making in this important group of patients.

## Abbreviations

APPROACH: Alberta Provincial Project for Outcomes Assessment in Coronary Heart Disease
AUROC: Areas under the receiver operator characteristic
CABG: Coronary artery bypass grafting
EuroSCORE: European System for Cardiac Operative Risk Evaluation
STS: Society of Thoracic Surgeons
